# Pesticide Pollution: Detrimental Outcomes and Possible Mechanisms of Fish Exposure to Common Organophosphates and Triazines

**DOI:** 10.3390/jox12030018

**Published:** 2022-09-02

**Authors:** Ihab Khatib, Piotr Rychter, Halina Falfushynska

**Affiliations:** 1 Department of Physical Rehabilitation and Vital Activity, Ternopil Volodymyr Hnatiuk National Pedagogical University, 46027 Ternopil, Ukraine; 2 Faculty of Science & Technology, Jan Dlugosz University in Czestochowa, Armii Krajowej 13/15, 42-200 Czestochowa, Poland; 3 Department of Marine Biology, Institute for Biological Sciences, University of Rostock, 18051 Rostock, Germany

**Keywords:** organophosphate pesticides, triazine pesticides, fish, accumulation, toxicity, adverse outcomes

## Abstract

Pesticides are well known for their high levels of persistence and ubiquity in the environment, and because of their capacity to bioaccumulate and disrupt the food chain, they pose a risk to animals and humans. With a focus on organophosphate and triazine pesticides, the present review aims to describe the current state of knowledge regarding spatial distribution, bioaccumulation, and mode of action of frequently used pesticides. We discuss the processes by which pesticides and their active residues are accumulated and bioconcentrated in fish, as well as the toxic mechanisms involved, including biological redox activity, immunotoxicity, neuroendocrine disorders, and cytotoxicity, which is manifested in oxidative stress, lysosomal and mitochondrial damage, inflammation, and apoptosis/autophagy. We also explore potential research strategies to close the gaps in our understanding of the toxicity and environmental risk assessment of organophosphate and triazine pesticides.

## 1. Introduction

Pesticides belong to the big group of chemicals used in various fields of farming, including pest control, prevention of agricultural production from losses, and controlling vector-borne disease (e.g., malaria, dengue) [1,2,3]. Currently, throughout the world, approximately 4 million tons of pesticides are utilized annually, and China, United States, Brazil, Argentina, Canada, Ukraine, France, Malaysia, Australia, and Spain belong to the top 10 pesticide-consuming countries, according to Worldometer data [4]. Moreover, China, Oceania, and the Americas had the highest growth rates in total pesticides use from the 1990s to the 2010s, while Europe was claimed to have the lowest rate up to 2018 [5]. The rapidly increasing amount of pesticides in use basically explains that 64% of global agricultural land is at risk of pesticide pollution by more than one active ingredient, and 31% is at high risk [3]. Moreover, pesticide pollution is tending to increase in the near future.

Due to their widespread usage, pesticides damage lakes and rivers through runoff from fields, and because of their capacity to bioaccumulate and disrupt the food chain, they pose a risk to animals and humans [6,7,8]. Fish represent an essential component of freshwater and marine ecosystems and play an important role in maintaining their balance [9]. While acute pesticide exposure might result in abrupt fish die-off, low-dose but long-term exposures can cause cellular and tissue damage, which happens to end up impairing health status and making them vulnerable to additional stressors. To elaborate, the exposure of Nile tilapia to 0.68 μg·L^−1^ bifenthrin for 60 days was reported to suppress immunorelated parameters, namely, lysozymes, IgM, IgG, and NO, and significantly reduced the resistance of fish against *Aeoromans* infection [10].

Fish are thought to be effective bioindicators of long-term toxic effects and a variety of habitat conditions due to their movement and relatively lengthy lifespan [9,11,12,13,14,15]. According to reports, several teleost and zebrafish have metabolic traits resembling those of humans, making them potential alternative candidates for mechanistic research of cellular events triggered by physical and chemical stimuli [15,16].

There is no pure specific mode of action by which pesticides can affect animals and humans. For instance, organophosphate pesticides are reported to affect acetylcholinesterase (AChE), an enzyme that hydrolyzes the neurotransmitter acetylcholine at neuromuscular junctions and brain cholinergic synapses. Because of that, organophosphates are extremely neurotoxic [17]. On the other hand, they are claimed to damage hormonal balance, provoke immune disorders, and cause oxidative damage to lipids, proteins, and DNA [2,7,18,19,20,21]. In terms of pesticide-induced toxicity in fish, recent papers have shown that biological effects comprise oxidative stress as the most common outcome of pesticide effects, in addition to neurological disorders, endocrine disorders, developmental toxicity, and metabolic changes. However, pesticide pollution is of secondary priority in the scientific community compared to other pollution. To elaborate, 23,358 articles appear in PubMed for the keywords “metal” and “fish”, but only 9133 for “pesticide” and “fish.”

The objective of this narrative review is to emphasize possible adverse effects and mechanisms of toxicity of commonly used organophosphate and triazine pesticides on fish species, revealing effects on biological redox activity, immunotoxicity, neuroendocrine disorders, and cytotoxicity. We also discuss the bioavailability, main routes of pesticides, their residual ingestion, bioaccumulation, and bioconcentration, and the shared toxicity mechanisms between organophosphate and triazine pesticide and other pesticides in aquatic organisms. Based upon our review, we identify some gaps in mechanistic studies as well as pesticides that have to be covered. Such data are expected to improve understanding of pesticide-induced cell damage in fish and extrapolate detrimental outcomes to mammals and humans.

## 2. Spatial Distribution of Common Pesticides in Water Bodies

The EU Pesticides Database in April 2022 counts 1471 active substances, with almost 600 of them not approved for use in the EU [22]. Meanwhile, the current list of pesticides that have been permitted for use in some countries in Eastern Europe is a bit longer. In particular, the Ukrainian pesticide database in 2022 has 2176 items [23].

Herbicides are the most commonly used pesticides for plant protection in Central and Eastern Europe. As mentioned in the Food and Agriculture Organization Corporate Statistical Database (FAOSTAT), herbicides, together with fungicides and insecticides (no more than 12%), contribute to more than 95% of the total pesticides in Europe [5]. In Ukraine, chlorpyrifos, imidacloprid, glyphosate, tebuconazole, and terbuthylazine belong to the most commonly used pesticides—18.1% of total pesticides [23]. This is consistent with the worldwide consumption of pesticides, at around 50% herbicides, 22% insecticides, and 15% fungicides [1,5]. Only Africa is an exemption, where insecticides and fungicides, such as penconazole, mancozeb, 2,4-dichlorophenoxyacetic acid (2,4-D), and chlorpyrifos, account for the majority of pesticides used [24].

The intensive application of pesticides to crop harvesting and plant disease control might lead to their increasing appearance in different environmental compartments, namely, soils, surface, and groundwater, because of the huge runoff from fields and urban areas. Unfortunately, the worst scenario has come true. It was shown that some Polish rivers are heavily contaminated with pesticides. In particular, the Warta River in West Poland was polluted with 22 pesticides (limit of quantification, LOQ > 0.005 μg·L^−1^) belonging to the phenylurea, sulfonylurea, neonicotinoid, chlorotriazine, triazine, and chloroacetanilide classes. The majority of the detected pesticides were herbicides (60%), followed by fungicides (24%) and insecticides (16%). Isoproturon, nicosulfuron, imidacloprid, terbuthylazine, chlorotoluron, S-metolachlor, and prometryn were found at the highest concentrations. Meanwhile, the essential thing is that their highest level (0.472 µg·L^−1^) and the hazard quotient (HQ) values did not exceed the permissible levels related to significant health risk [25]. Similar findings were also reported by Bojakowska et al., who highlighted that 95% of the analyzed river sediment samples picked up from Polish rivers, namely, Vistula, Warta, Bug, Narew, and Oder, and about 90% of analyzed lakes were contaminated with polychlorinated biphenyls [26,27]. The most frequently detected pesticides were isomer γ-HCH (lindane) and the dichlorodiphenyltrichloroethane (DDT) group compounds (up to 8.51 µg·kg^−1^ [26]. The maximum lindane concentration in studied lakes was 60.7 ng g^−1^, that of DDTs 602.9 ng·g^−1^, and of polychlorinated biphenyls (PCBs) 50.7 ng·g^−1^, while the averages were 6.3, 19.2, and 2.8 ng·g^−1^, respectively [27].

A similar state of play is in Germany and Hungary. As previously shown, the principal contaminants of the Elbe River include atrazine, terbuthylazine, metazachlor, metolachlor, isoproturon, and chlorotoluron [28]. Atrazine is a herbicide that has been discovered in 669 samples over a lengthy period of time, with concentrations above the LOQ in 78% of all samples of the Elbe River. Atrazine and terbuthylazine, which have lately replaced atrazine, had high maximum values of 270 and 340 μg·L^−1^, respectively [28]. In Hungarian water bodies, atrazine levels were found to be slightly lower. It was detected at levels of around 100 ng·L^−1^ in the majority of cases [29]. Hungary was historically better renowned for capturing hotspots, such as the Balatonfűzfő region. In several cases, atrazine levels in surface and groundwater reached 8240 and 7540 ng·L^−1^, respectively [29]. Terbuthylazine is also one of the most dangerous contaminants in the Hungarian river system. terbuthylazine, S-metolachlor, and tebuconazole had the highest time-weighted average concentrations among pesticides detected in the Drava River, one of Central Europe’s largest and most important rivers, during 2018–2021, reaching 439,83 (~23.5 ng·L^−1^), 1140, and 19.7 ng/sample, respectively. Some chlorophenoxy acids (8.8–18.2 ng/sample) were also found at lesser levels (2,4-D, mecoprop-P, and 2-methyl-4-chlorophenoxyacetic acid-MCPA) [30].

Despite the fact that European rivers and lakes are contaminated with pesticides, the overall amount of pollution is significantly lower than in the United States, South Africa, and South America. Terbuthylazine (71.8–717.0 ng·L^−1^), acetamiprid (11.2–111.7 ng·L^−1^), chlorpyrifos (0.4–4.2 ng·L^−1^), thiacloprid (11.5–115.2 ng·L^−1^), and imidacloprid (726.0–7249.4 ng·L^−1^) in South African river samples exceeded Environmental Quality Standards values up to 3-, 5-, 9-, 12-, and 558-fold, respectively [24]. In addition, in the freshwater vicinities of the Amazon River, 11 pesticides, including organophosphates, benzimidazole, and triazines, were found. Chlorpyrifos, carbendazim, diuron, atrazine, and terbuthylazine had the highest prevalence. With a maximum concentration of 535 ng·L^−1^, malathion had the highest overall concentration of insecticides. For herbicides, most exposure amounts were moderate, with the exception of metolachlor, atrazine, and terbuthylazine, which had concentrations up to 25–28 ng·L^−1^ in certain streams around Manaus. The samples taken from the Amazon River, the Negro River, one stream in Macapá (186 ng·L^−1^), and one stream in Belém (100 ng·L^−1^) all had the greatest bulk quantities of chlorpyrifos, while chlorpyrifos-methyl was discovered at relatively low concentrations [31]. To continue with pesticide pollution in Brazilian water bodies, it has to be mentioned that 97% of the studied municipalities displayed pesticide levels that were much higher (189.84 ppb) than the EU-set limits (0.5 ppb) [32].

In the case of terbuthylazine, the lowest detected value in South African rivers was three times higher than the time-weighted average concentrations in the Danube basin [24,30] and the highest measured concentrations in Smederevo and the Danube tributaries Morava and Tisa (in 2010–2011 200 ng·L^−1^, 180 ng·L^−1^, and 130 ng·L^−1^ respectively) [33].

Glyphosate and chlorpyrifos, which have lately been prohibited in various nations, are receiving a lot of attention these days. The United States, Germany, and the United Kingdom, for example, have indicated that they will phase out the use of glyphosate and chlorpyrifos in the near future [34]. However, the global glyphosate market is projected to reach $8.9 billion by end of 2026 and will have tended to increase by 2027 [35]. Despite the fact that glyphosate sales are expected to rise, little is known about the average glyphosate or its metabolite aminomethylphosphonic acid (AMPA) content in Central and Eastern European water bodies. The average glyphosate concentrations in 66 drinking water samples collected at random from different water treatment plants around Greater Poland were in the range of 0.07–0.31 µg·L^−1^, a thousand times lower than the acceptable daily intake [36]. Glyphosate concentrations in river Rhine samples were all between 25 and 55 ng·L^−1^, while AMPA values were between 55 and 65 ng·L^−1^ [37]. Glyphosate concentrations in the hypolimnia of Lake Murtensee and Lake Greifensee were around 15 and 35 ng·L^−1^, respectively. In both lakes, AMPA concentrations were slightly higher, with levels about 60 ng·L^−1^ [37]. Canada and Switzerland had the lowest glyphosate amounts in surface water among the mentioned nations (0.025–0.042 μg·L^−1^) [38,39,40]. Meanwhile, several European countries, such as Italy and France, are thought to have slightly greater glyphosate quantities in their water bodies. Maximum glyphosate concentrations in water samples were 260 nanograms per liter, 15 ng·L^−1^ in sediment, and 7 ng·L^−1^ in suspended particulate matter collected from the seven sampling locations in Venice lagoon [41]. Glyphosate and AMPA are two of the most commonly detected chemicals in rivers in France, with quantities reaching up to 164 and 558 μg·L^−1^ for glyphosate and AMPA, respectively [42].

With rare exceptions, glyphosate/AMPA concentrations in Europe are substantially lower than in the United States and South America, according to available data. In particular, glyphosate and AMPA were detected in 74% and 90% of US stream samples from 2015 to 2017 and maximum values were 6.08 and 5.07 μg·L^−1^, respectively, up to 20-fold that in Europe [43]. There were maximum annual time-weighted mean concentrations of 1.39 and 2.55 μg·L^−1^ for glyphosate and AMPA, respectively [43]. A similar situation is in Argentina, which has been accused of using huge amount of pesticides, including 240,000 tons of glyphosate per year. This has resulted in glyphosate and AMPA being detected in 100% of samples of studied water bodies and soils [44]. The levels of glyphosate in waters ranged from 0.10 to 0.70 mg·L^−1^, while in sediments and soils values were between 0.5 and 5.0 mg·kg^−1^. Glyphosate also had the highest concentrations among all the matrices studied (3868 ppb), exceeding by far the other pesticides: endosulfan II (337.7 ppb) and chlorpyrifos (242 ppb) [44,45]. The highest rate of detection was found in stream sediment samples (glyphosate 95%, AMPA 100%), followed by surface water (glyphosate 28%, AMPA 50%) and then groundwater (glyphosate 24%, AMPA 33%) [46].

Commonly found in fruit, vegetables, and drinking water, chlorpyrifos is the elephant in the room regarding its appearance in European watersheds. All of this occurs in the context of chlorpyrifos prohibition in many countries, including the EU Member States, who were required to revoke all plant protection product authorizations containing chlorpyrifos and chlorpyrifos-methyl by 2020 [47]. It has recently been reported that chlorpyrifos in the range of concentrations 0.18–36.17 ng·g^−1^ d.w. sediments was the most abundant of nine detected pesticides in the Ebro River (Spain). Water samples from the same localities were also enriched with chlorpyrifos, up to 16.40 ng·L^−1^ [48]. The level of chlorpyrifos in Ebro River was in the same range as detected in the St. Lawrence River (Canada), <0.006 µg·L^−1^ [49]. However, in other places in Canada, namely, Environment Canada Pacific and Yukon Region and Ontario Region, chlorpyrifos was more ubiquitous. A total of 300 surface water samples were taken and chlorpyrifos was detected in around 40% of these samples. Concentrations ranged from the detection limit to 0.205 µg·L^−1^ [49]. A similar state of play is in Chile, New Zealand, and South Asia. Notably, chlorpyrifos was discovered in only 30% of the examined samples at the end of the summer season in Las Cabras Canal (Chile), with a highest value of 0.183 ± 0.065 µg·L^−1^ [50], when all other pesticide concentrations were below 20 ng·L^−1^. In the most polluted Chilika Lake and Tapi River (India), concentrations were 2.73 and 0.27 µg·L^−1^ respectively [51].

To summarize, pesticide levels in Eastern and Central European water bodies are relatively high, particularly in agricultural countries. Despite the fact that European countries are accused of polluting surface and groundwater with pesticides, North and South America, Asia, and South Africa are taking the lead. Organophosphates (e.g., chlorpyrifos, glyphosate), phenylureas (e.g., chlorotoluron), triazines (e.g., terbuthylazine), and neonicotinoids belong to the most commonly used and detected pesticides in water bodies across the world. Actual pesticide pollution is a worldwide problem and powerful enough to cause harmful disruption effects in non-targets, such as water animals and then humans. We must work to ensure that pesticide concentrations and residues are regularly monitored, as well as identify potential early warning biomarkers that will aid in determining the detrimental effects of xenobiotics on ecosystems and humans in the early stages, while full recovery is still possible.

## 3. Bioaccumulation of Organophosphate and Triazine Pesticides in Fish

Being amenable to bioaccumulation in living organisms, pesticides, even in background concentrations in the water column or sediments, might be accumulated in fish tissue [52]. The accumulation of pesticides in aquatic animals can be realized via two main routes, namely, direct pesticide uptake from water and uptake via contaminated food [53]. It has been shown that the rate of organophosphate pesticide accumulation is greater in freshwater fish than in marine fish [54]. In addition, mollusks are less susceptible to pesticide accumulation than fish, which is the opposite of metal accumulation [54].

Pesticide lipophilicity/hydrophobicity and log *K*_ow_ (n-octanol–water partition coefficient) are among the most important factors that determine the rate of pesticide accumulation in living organisms. In general, log *K*_ow_ < 3 features pesticides that are not fat-soluble, log *K*_ow_ = 3–4 classifies pesticides with lapping region, and log *K*_ow_ > 4 proves that the residues of pesticides defined as fat-soluble. In addition, if log *K*_ow_ > 4, they are accumulated almost in the fat fraction [55]. It is worth mentioning that the log *K*_ow_ value of chlorpyrifos is 4.9, which allows us to consider chlorpyrifos as the bioaccumulative substance in animal tissue [56]. On the other hand, highly toxic and persistent triazine pesticides, namely, simazine (log *K*_ow_ = 2.3), atrazine (log *K*_ow_ = 2.7), and terbuthylazine (log *K*_ow_ = 3.4) as well as malathion (log *K*_ow_ = 2.18 ÷ 3.06) and particularly commonly used organophosphate pesticide glyphosate (log *K*_ow_ = −1.7 ÷ 1.67) have lower log *K*_ow_, which highlights low-affinity accumulation inside the cells [57,58]. Once accumulated, chlorpyrifos and atrazine are excreted mainly through the kidneys [59]. Being processed in an organism, their hydroxylated, N-dealkylated, oxidized, and conjugated metabolites, including 3,5,6-trichloro pyridine-2-phenol (TCP), diethylphosphate, and diethylthiophosphate, are eliminated in the urine [59]. It was worth noting that atrazine metabolites are considerably less hazardous than the parent molecule and are typically absent from the environment in concentrations that could be harmful to living organisms [60].

It is expected that bioconcentration factor (BCF), particularly log BCF, and log *K*_ow_ have to be in a linear relationship. However, physiological peculiarities of the very species can affect the uptake, metabolism, and elimination of a pesticide and explain the deviation of the BCF value from the predicted one [61]. Because of this, the rectified equations were proposed for organophosphate pesticide accumulation in mollusks based on the reference data: log BCF = 0.543 log *K*_ow_ − 0.436 (*r*^2^ = 0.438, *n* = 17) and log BCF = −0.106 (log *K*_ow_ − 5.66)^2^ + 2.11 (*r*^2^ = 0.471, *n* = 17) [61].

It has been recently reported that three fish species, namely, mullet (*Liza auratam*), Caspian white fish (*Rutilus kutum*), and *Cyprinus carpio*, from five estuaries along the Caspian Sea (Iran) were contaminated with diazinon, malathion, and azinfos methyl. Their respective concentration ranges were determined as 0.01–0.16, 0.01–0.15, and 0.05–0.36 mg·kg^−1^ and they were marginally lower than the permissible level for edible fish [62]. In addition, fish *Jenynsia* *multidentata* were able to accumulate chlorpyrifos in their tissue in the following order: intestine > liver > gills, and BCFs were in a range from 133 to 212 L·kg^−1^, which highlights the capacity of chlorpyrifos to be bioconcentrated in fish tissue [63]. In contrast to later findings, however, a considerably higher level of chlorpyrifos (0.463 mg·kg^−1^) had been detected in Taiwanese farmed fish between 2002 and 2004 [64]. Notably, the tissue concentrations of organophosphate pesticides in Asian sampling localities were commensurable with organochlorine ones. As an example, the mean concentration of DDT, hexachlorcyclohexan, endosulfan, chlordane, and hexachlorobenzene in all fish and mollusk samples from Liaoning province, China in 2007 was 15.41, 0.84, 1.31, 1.05, and 0.63 mg·kg^−1^, respectively [54].

The bioaccumulation (BAF) or even bioconcentration (in the case of trophic chains) factors pertain to the meaningful indices of pesticide accumulation in living organisms. However, the BAF/BCF values significantly vary among close related species, between results of field and laboratory experiments, and in vivo/in vitro conditions. In addition, BCFs of commercial formulation of pesticides are higher when compared to a main active ingredient. This finding reflects those of Bonansea et al., who found that the BCF of chlorpyrifos in the liver of *Jenynsia multidentata* fish exposed to a commercial pesticide formulation that contains adjuvants was higher than the value obtained in fish exposed to chlorpyrifos individually [63]. The question raised by this is whether the application of some prospective adjuvant that decreases uptake of a pesticide by non-targets can help attenuate its expected toxicity to living organisms. Such a trend is very prospective in cancer therapy as a promising strategy to enhance anticancer drug effectiveness via cell membrane permeability [65,66].

When we dig deeper into the comparative analysis of BCF and its dependents from predictors, we can see that the data regarding BCF vary significantly. In particular, the bioconcentration factor of chlorpyrifos calculated for omnivorous planktivorous killifish *Jenynsia* *multidentata* varied from low values (25  ±  16 and 35  ±  19 in muscle and gills, respectively) to high values (1024  ±  593 in liver) [63]. Comparable BCF values for chlorpyrifos were reported for other fish species. As an example, log BCF of chlorpyrifos in fathead minnow (*Pimephales promelas*), guppies (*Poecilia reticulata*), mosquitofish (*Gambusia affinis*), killifish (*Oryzias latipes*), goldfish (*Carassius auratus*), and zebrafish (*Danio rerio*) were in the range of 2.7–3.84 [67,68,69,70,71]. Meanwhile, calculated BCF for other omnivorous fish, *Cyprinus carpio*, ranged from 3 to 10 in gills and liver tissue after 96 h of animal exposure with 0.03–0.1 μg·L^−1^ chlorpyrifos, which is up to 100 times lower than reported in the abovementioned cases [72].

Not too many organophosphate pesticides have a stronger tendency to be accumulated in living organisms than chlorpyrifos. Among them is diazinon, which was among the three most detectable pesticides in fish and mollusks from the Seto Inland Sea in Japan (2016–2017). Its BCF values reached 9.125 L·kg^−1^ for fish and marine animals [73]. The highest hazard quotients (HQ = 0.0034–0.2196) for diazinon had determined in red sea bream and greenling indicated a medium risk to consumers when HQ was 0.1 < HQ < 1.0 [73,74].

Lower than chlorpyrifos BCF was found to be for some organophosphate pesticides, tetrazine pesticides, and novel polycyclic pesticides. In particular, log BCF of malathion was in the range of 0.65 to 2.01 for edible fish [75]. Log BCF values of 0.6 and 1.17 were experimentally determined in zebrafish for atrazine at 100 and 500 μg·L^−1^, which proved that bioaccumulation of atrazine is limited [71]. The novel cyclic organohalogene insecticide broflanilide showed a medium bioconcentration factor value of 10.02 and 69.40 in *Danio rerio* at 2.00 and 0.20 mg·L^−1^, respectively [76]. Therefore, marine and particularly freshwater fish absorb chlorpyrifos and diazinon in their bodies and may be affected because of pesticide potency to cause adverse effects on biota. When they occur together in bodies of water, they can promote additive toxicity [77]. All of that increases the hazard of pesticide pollution.

Mollusks are known to accumulate huge amounts of metals, although they are claimed to accumulate a lower level of pesticides than fish [78]. It has been reported that the BCF value in *Mytilus edulis* after exposure to 3.2 mg·L^−1^ of chlorpyrifos was 482 + 86, which was approximately twofold less than reported in fish (see the abovementioned information) [79]. In natural land-based ponds in Taiwan, only 0.18% of all freshwater clams accumulated chlorpyrifos and trichlorfon in their tissue at the maximum levels of 0.05 and 0.02 mg·kg^−1^ [80]. The average value of BAF for seven organochlorine pesticides, including HCHs, DDTs, and heptachlor, in mollusks from the Soai Rap estuary, Ho Chi Minh City, was determined to be 56.672 in *Crassostrea gigas*, 66.730 in *Perna viridis*, 123.884 in *Anadara granosa*, 52.060 in *Meretrix lyrata*, and 115.176 in *Pinctada maxima* [81]. The findings reported here provide a deeper insight into pesticide control and highlight the validity of fish as more sensitive bioindicator species for biomonitoring activities due to their higher ability to accumulate pesticides.

To sum up, pesticide lipophilicity and log *K*_ow_ belong to the most important factors that determine the rate of pesticide accumulation in living organisms. Species specificity, experimental conditions, and presence of additives in pesticide formulation should be taken into account to omit spurious predictions or even conclusions regarding BAF/BCF. Chlorpyrifos among organophosphates, triazine, and organochlorine pesticides possess the highest ability to be accumulated in water animals.

## 4. Organophosphate and Triazine Pesticides: Adverse Effects for Fish

Pesticides and their residues, if entering living organisms, can have long-term negative impacts on animals and humans in particular, as well as ecosystems in general [82,83,84]. It has been recently mentioned that the level of water pollution by pesticides was positively correlated with the total burden of cancer cases in the population of Parana State, Brazil, emphasizing pesticide pollution matters [32]. Variation in bioaccumulation, lipophilicity, potency of a target metabolite/enzyme affection, and chemical groups or adjuvants modified the basic structure of pesticides resulting in varying poisoning onset, severity of an injury, and clinical toxidrome [85].

The most common mechanism for organophosphates is to inhibit AChE by phosphorylating the serine residue within the enzyme active site. Inhibition of AChE causes an increase in the amount and time of acetylcholine residence at nicotinic and muscarinic receptors and then acetylcholine accumulation and hyperstimulating cholinergic receptors, resulting in a clinical toxidrome known as “cholinergic crisis,” which can cause metabolic imbalance. It has been emphasized, however, that only a small number of organophosphate pesticides among 51 studied are able to bind directly to cholinesterases in the parent form [17].

Not only are organophosphates known to affect organisms but also their bioactive metabolites after processing by liver cytochromes P450. Bioactive metabolites are reported to suppress serine active-site enzymes, among them esterases (B-esterases, carboxylesterase), proteases, hydrolases (fatty acid amide hydrolase), and lipases (e.g., lysophospholipase). The formation of the covalent bond between the alkyl phosphorus group of an organophosphate compound and the active-site serine’s hydroxyl residue is what causes organophosphates to block serine hydrolases [86,87].

Apart from neurotransmitters, other molecular and cellular targets, including neurotrophic variables, hormones, the immune system, enzymes involved in the breakdown of β-amyloid protein, and proinflammatory cytokines, belong to the molecular targets of organophosphates [2,18,21]. As a result, organophosphates are thought to cause neurological diseases, behavioral complications, respiratory stress, metabolic disorders, including alteration in carbohydrate degradation pathways, oxidative stress, DNA damage, immune and endocrine disorders, mitochondrial toxicity, inflammation, and delayed metamorphosis [88]. Initially, prominent changes in cell processes and stress responses are associated with histopathological disorders, such as regressive, vascular, and progressive gill disorders, swelling in chloride cells, and necrosis [89,90]. It is worth noting that triazines possess similar signs of toxicity in animal models with special emphasis on DNA damage and endocrine disorders [91].

### 4.1. Oxidative Stress as the Dominant Non-Cholinesterase-Related Downstream Adverse Effect of Organophosphate and Triazine Pesticides

Biological systems have evolved appropriate antioxidant mechanisms to protect their cells and compartments from oxidative damage that can occur as a result of detrimental reactive oxygen species (ROS) effects on biopolymers and cells [92]. Some xenobiotics, particularly pesticides, toxic metals, nanooxides, and pharmaceuticals, can cause a discrepancy between endogenous and external ROS, resulting in a reduction in antioxidant defense [93,94]. When the physiological equilibrium between the ROS overproduction and their neutralization by antioxidants is disrupted, ROS accumulate in cells, causing oxidative stress and tissue damage [92]. The more end products of lipid peroxidation, protein carbonylation, nucleic acid fragmentation, and mitochondrial impairment accumulate in cells, the faster they are committed to programmed cell suicide. The oxidative stress in general and lipid peroxidation and protein carbonylation in particular are involved in various pathological states, including inflammation, degenerative illnesses, and aging.

ROS are regarded to be the main cause of oxidative stress. Only 1–5% of ROS formed during the process of cellular respiration may trigger oxidative destruction of biomolecules. The most common radicals are the hydroxyl radical (^•^OH), superoxide anion radical (O2^•−^), hydrogen peroxide (H_2_O_2_), singlet oxygen (^1^O_2_), nitrogen (II) oxide (NO), peroxynitrite (ONOO^−^), alkoxyl (RO^•^), and the peroxide radical (ROO^•^) [92,94]. The most reactive of these is the hydroxyl radical ^•^OH, which can be produced via the Fenton reaction and the Haber–Weiss reaction from hydrogen peroxide and redox-active metals, namely, iron and copper [95]. Direct detection with electron spin resonance, microdialysis, measurement of the free radical by a trapping agent, as well as indirect measurement of oxidized products, such as lipid peroxidation and DNA damage, have all been employed to explore the cellular formation of ^•^OH [96].

**Figure 1:**
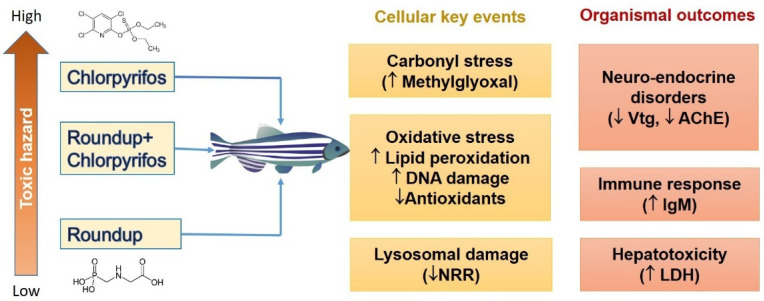
Cellular key events and organismic adverse outcomes in zebrafish (*Danio rerio*) from the effects of organophosphate pesticides (Roundup and chlorpyrifos) (Reprinted with permission from [19]. 2022, Elsevier).

Malondialdehyde (MDA), one of the end products of lipid peroxidation, has been explored the most in water animals. A number of large cross-sectional studies have found that MDA/thiobarbituric acid-reactive substance (TBARS) levels in water animals rise after exposure to a variety of pesticides, particularly organophosphates (Table 1). For example, after 96 h of exposure to the commercial formulation Colosso FC30 containing chlorpyrifos (0.5 or 1 μg·L^−1^) and cypermetrin (0.3 or 0.6 μg·L^−1^), both carp and zebrafish demonstrated an increase in lipid peroxidation [97]. In the case of prolonged exposure, similar outcomes were obtained. *Danio rerio* treated with chlorpyrifos (0.1 µg·L^−1^ and 3 µg·L^−1^) and Roundup (15 µg·L^−1^ and 500 µg·L^−1^) for 14 days (Figure 1), *Ctenopharyngodon idellus* fish treated with 1.4 and 2.44 µg·L^−1^ chlorpyrifos for 15, 30, and 60 days, as well as yellow-tailed tetra fish (*Astyanax altiparanae*) treated with 1 and 2 µg·L^−1^ atrazine for 30 days, showed significant increases in lipid peroxidation in the liver, kidney, and gills [19,98,99]. The latter event was in good agreement with an increase in ROS production on the one hand and a drop in catalase, superoxide dismutase, and glutathione peroxidase (GPx) activity on the other [98].

Lipid peroxidation (LPO) is especially vulnerable to acute pesticide exposure since it is induced after environmentally relevant pesticide exposure. Its variability index has reached more than 5 in some cases. There are numerous published studies describing the activation of lipid peroxidation in fish following acute exposure to organophosphates (e.g., Roundup, malathion), triazines (atrazine), and pyrethroid pesticides (deltamethrin, cypermethrin, and lambda-cyhalothrin) [100,101,102]. Acute (10 mg·L^−1^) treatment of *Prochilodus lineatus* with Roundup for 96 h resulted in a threefold elevation in MDA in the fish liver [100]. LPO levels were also found to be higher in the gills and plasma of *Catla catla* following 96 h of exposure to acute (0.09 ppm) and sublethal (0.009 ppm) concentrations of methyl parathion. Significant changes in LPO were found to be in good agreement with the alterations in antioxidants and metabolic enzymes, such as glutamic oxaloacetic transaminase (GOT), glutamic pyruvate transaminase (GPT), and lactate dehydrogenase (LDH), which could have been caused by severe cellular and tissue damage [103]. When tropical *Astyanax aeneus* fish were exposed to a sublethal concentration of ethoprophos (0.01 mg·L^−1^ for 48 h), LPO increased, though catalase (CAT), glutathione S-transferase (GST), and resting metabolic rate were unaffected [104].

When animals are exposed to an acute pesticide for an extended period of time, the severity of the harm increases. Rainbow trout exposed to acute glyphosate-based herbicide (1.25 mg·L^−1^ and 2.5 mg·L^−1^) for 14 days showed a significant increase in total oxidant levels as well as prominent histopathological disorders, including irregular or even degenerated secondary lamellae and epithelial hyperplasia in gill sections, swelling in chloride cells, and necrosis [90]. On the basis of notable (up to 4.4 times) LPO elevation in liver tissue, the significant effect of acute exposures of the freshwater air-breathing fish *Channa punctatus* to atrazine in concentrations of 4.238–10.600 mg·L^−1^ for 15 days has been determined. Simultaneously with LPO induction, there was a considerable decrease in CAT activity, which could be attributable to a decrease in response rate as a result of excess H_2_O_2_ generation [101]. Meanwhile, common carp were given triazine metabolites (terbuthylazine 2-hydroxy—0.73 μg·L^−1^; terbuthylazine-desethyl −1.80 μg·L^−1^; atrazine 2-hydroxy—0.66 μg·L^−1^) in much lower, environmentally relevant concentrations and showed no signs of oxidative stress or negative effects on hatching, behavior, embryo viability, growth, or early ontogeny [105]. In general, the increase in lipid peroxidation indicated above could be linked to uncompensated ROS formation in cells and a weakened antioxidant system in fish. ROS and oxidative stress have been shown to be apoptosis triggers, causing damage to proteins and lipids, as well as activating apoptotic pathways and/or the initiation of inflammatory processes.

ROS happen to be a significant cause of DNA damage, which, if not repaired properly, can result in mutations and chromosomal aberrations. Malondialdehyde and 4-hydroxynonenal, the most genotoxic and mutagenic lipid peroxidation products, form adducts with DNA, causing DNA damage [106]. Organophosphate pesticides may also cause DNA damage in terms of base alkylation, either directly or indirectly through protein alkylation. In particular, monocrotophos in low nanomolar concentrations after short-term exposure provoked an increase in tail DNA and micronuclei frequency in zebrafish in a dose-dependent manner [107]. In addition, glyphosate-based pesticides in subacute concentrations and chlorpyrifos, even in environmentally relevant concentrations, led to enhanced DNA damage signs in *Danio rerio* and *Anguilla anguilla*, appearing as DNA strand breaks and a level of oxidized purines in DNA [19,106]. Similar findings were proven to appear in agricultural workers exposed to pesticides while working [108].

Early life-stage organisms are more prone to pesticide impacts than adult counterparts because they are more fragile. It was recently shown that 3 h exposure of postfertilization (hpf) zebrafish embryos to chlorpyrifos (236 μg·L^−1^), beta-cypermethrin (5.9 μg·L^−1^), and a mix of them for 96 h resulted in four to eight times increased lipid peroxidation and marginally decreased antioxidant activity. Chlorpyrifos and beta-cypermethrin, individually and in combination, were also able to cause liver lesions and promote tissue death. The toxicity of these two pesticides was linked to extracellular matrix-receptor interaction, focal adhesion, cell cycle, DNA replication, phototransduction, and adherens junction pathways, according to transcriptome sequencing [109].

Many researchers are working on the issue of alleviating the adverse outcomes caused by pesticides these days, and antioxidants and immunostimulants are among the most promising [110,111,112]. MDA magnification was observed in *Oreochromis niloticus* treated with 2 mg·L^−1^ Roundup and 0.5 mg·L^−1^ malathion for 60 days, which was successfully alleviated by selenium yeast addition (3.3 mg·kg^− 1^ available selenium) [112]. In *Nile tilapia*, an Isatis supplemented diet (1%) alleviated atrazine-induced growth retardation, hepatorenal dysfunction (uric acid, urea, creatinine, aspartate transaminase—AST, alanine transaminase—ALT, as well as total protein, globulin, and albumin) and oxidative stress (measured by MDA, SOD, GPx, and CAT) caused by 1.39 mg·L^−1^ exposure for 30 days [111]. Similar results were found in *Nile tilapia* exposed to chlorpyrifos (15 μg·L^−1^) after spirulina crude extracts (0.5% and 1%) administration. Fish fed a spirulina-supplemented diet had higher antioxidant enzyme activity, such as SOD and CAT, and lower aminotransferases and alkaline phosphatase (ALP) activities, which emphasized both the preventive and restorative effects of spirulina against chlorpyrifos toxicity [113].

Importantly, the research findings on pesticides’ ability to cause oxidative lipid damage in non-target water animals, i.e., fish, are consistent with human findings. Acute in vitro exposure to terbuthylazine (8 ng·mL^−1^) resulted in a significant increase in lipid peroxidation and low-level DNA instability in human blood samples. Specifically, DNA migration obstruction was linked to DNA cross-links caused by reactive terbuthylazine metabolites, rather than oxidative DNA damage [91]. In addition, the findings disclosed by Intayoung et al. suggested that using glyphosate and paraquat together resulted in a significant increase in urinary MDA levels [114]. It is possible, therefore, that pesticides, by causing oxidative stress in non-target organisms, may be linked to profound adverse health effects, including cancer, not only in animals but also in humans, and early-warning hazard assessment using fish as an alternative model can provide plenty of benefits.

Therefore, there is significant evidence from fish studies to suggest that exposure to organophosphates and triazines, even in low realistic environmental concentrations, can result in profound oxidative stress and oxidative injury. However, more efforts need to be made to shed light on the molecular mechanisms of pesticide-driven adverse effects in fish, particularly for triazines. Antioxidant and immunomodulatory food supplements, including plant-derived ones, might mitigate the adverse outcomes caused by pesticides.

**Table 1:** Biochemical, physiological, and cytological alterations in fish after the effects of commonly used organophosphate pesticides.

Species	Pesticide, Concentration	Effects	Reference
Chlorpyrifos exposure			
Common carp (*Cyprinus carpio*)	Colosso FC30, (0.3 μg·L^−1^ CYP +0.5 μg·L^−1^ CPF; 0.6 μg·L^−1^ CYP + and 1 μg·L^−1^ CPF	TBARS↑, NPSH↓, ASA~, GST↑, CAT↓	[97]
Common carp (*Cyprinus carpio*)	0.1, 0.05, 0.03 μg·L^−1^ CPF in combination with 0.0006, 0.0003, 0.0001 μg·L^−1^ CYP correspondingly, 96 h	CAT↑, GR↓, GPx↓	[72]
Zebrafish (*Danio rerio*)	Colosso FC30, (0.3 μg·L^−1^ CYP +0.5 μg·L^−1^ CPF; 0.6 μg·L^−1^ CYP + and 1 μg·L^−1^ CPF	TBARS↑, NPSH↑, ASA↓, GST↑, CAT~	[97]
Zebrafish (*Danio rerio*) early life stages	CPF (236 μg·L^−1^), CYP (5.9μg·L^−1^) and 236 μg·L^−1^ CPF + 5.9 μg·L^−1^ CYP, 96 h	Malformation, death in larvae, affected hatchability SOD↓, MDA↑, CAT(CPF) ↓, apoptosis ↑	[109]
Zebrafish (*Danio rerio*)	Chlorpyrifos, 0.1 µg·L^−1^ and 3 µg·L^−1^, 14 days	Mitochondria swelling↑, Lysosomal stability↓, Cathepsin D↑, LDH↑, SDH↓, Methylglyoxal↑, ROS↑, RNS↑, TAC↓	[115]
Carp, *Ctenopharyngodon idellus*	Chlorpyrifos, 1.4 µg·L^−1^ and 2.44µg·L^−1^ for 15, 30 and 60 days	SOD↓, MDA↑, CAT~, GSH↓, GST↓	[98]
Rainbow trout, *Oncorhynchus mykiss*	Chlorpyrifos, 0, 2, 4 and 6 μg·L^−1^ 7, 14, 21 days	erythrocyte count ↓, haemoglobin↓, haematocrit↓, leucocyte count↑, histological disturbances↑, AChE↓, short-term: SOD↑, CAT↑; long-term: SOD↓, CAT↓	[116]
Largemouth bass (*Micropterus salmoides)*	4 μg·L^−1^ CPF, 60 days	Apoptosis ↑, Inflammation ↑, MDA↑, SOD↑, GPx~, ACP~, CAT~, ALP~, *Nrf2*↑, *cat*~↑, *sod*~↑	[117]
Zebrafish (*Danio rerio*) early life stages	100 and 300 μg·L^−1^ CPF, 96 hpf	Hatchability↓, heart rate↓, morphological abnormalities↑, Immunotoxicity↑, MDA↑, CAT~, GSH↓, TNFα↑, IFN↑, IL-1β↑, IL6↑, C4↑	[118]
Glyphosate/Roundup exposure			
European eel (*Anguilla anguilla*)	58, 116 μg·L^−1^ (Roundup), 1 and 3 days	DNAsb↑, erythrocytic nuclear abnormalities↑, LPO↑ CAT~, GST~, GPx~, GR~, GSHt~	[119]
Spotted snakehead (*Channa punctatus*)	3.25–6.51 mg·L^−1^ (Roundup) 1, 7, 14, 21, 28, 35 days	TBARS↑, DNA damage↑, LPO↑, ROS; CAT↓, SOD↓, GR↓	[120]
Goldfish (*Carassius auratus*)	2.5–20 mg·L^−1^ (Roundup), 2 month	CAT↑; GSH↓, GST↓, GR↓, G6PDH↓, SOD↓	[121]
Goldfish (*Carassius auratus*)	0.2 mmol·L^−1^ (Nongteshi, 30% glyphosate), 90 days	creatinine ↑, urine nitrogen↑, ALT↑, AST↑, LDH↑, MDA↑, 3- hydroxybutyrate↑; SOD↓, GPx↓, GR↓	[122]
Air-breathing teleosts *Anabas testudineus* *Heteropneustes fossilis*	17.20 mg·L^−1^ (Excel Mera 71)	AChE↑, LPO↑, CAT↑; GST↓, Total protein↓	[123]
Zebrafih (*Danio rerio*)	10, 50, 100, 200, 400 μg·L^−1^ (Glyphosate), 48 h	Nitric Oxide↓, Cacana1C↓, RYR2a↓, HSPb11↑	[124]
Malathion exposure			
Stinging catfish (*Heteropneustes fossilis*)	0.44, 0.88 and 1.76 mg·L^−1^, 3 weeks	Erythrocytes↓, Leucocytes↓, Hemoglobin↓, Ht↓, Glucose↑, Plasma proteins↓, glutamic-oxaloacetic transaminase↑, glutamic-pyruvic transaminase↑	[125]
Zebrafish (*Danio rerio*)	5 and 50 µg·L^−1^, 14 days	Mito swelling↑, Lysosomal stability↓, Cathepsin D↑, LDH↑, SDH↓, Methylglyoxal~, ROS~, RNS~↑, TAC↓↑	[115]
*Spotted snakehead (Channa punctatus)*	0.4 mg·L^−1^, 1, 4, 8, 12 days	SOD↑, CAT↑, LPO↑, serum glucose↓, protein↑, cholesterol~, albumin~	[126]
*Black pacu* (*Colossoma macropomum*)	7.30 mg·L^−1^, 96 h	Mitochondrial respiration~, ROS (mito)↓, GST↑, CAT↑, SOD↑, RAS↑, LPO~, p53~	[127]
Senegalese sole (*Solea senegalensis*) early life stages	1.56, 3.12, and 6.25 μg·L^−1^	AChE↓, BChE↓, CbE↓, pyknotic nuclei↑, CYP1A~, expression AChE~	[128]
Pesticide toxic effects + dietary supplement			
Nile tilapia (*Oreochromis niloticus*)	15 μg·L^−1^ CPF, 28 days CPF + 0.5% and 1% *Spirulina platensis*, 28 days	CPF: ALT↑, AST↑, ALP↑, SOD↓, CAT↓, MDA↑ CPF + *Spirulina*: ALT↓, AST↓, ALP↓, SOD↑, CAT↑, MDA↓	[113]
African sharpthooth catfish (*Clarias gariepinus*)	8.75 μg·L^−1^ CPF, 6 weeks CPF + Carica papaya (250 mg·kg^−1^ bw),6 weeks	CPF: ALT↓, AST↑, Glucose↑, AChE↓, TAC↑, MDA↑, GSH↓ CPF + *papaya*: ALT↑, AST↓, Glucose↓, AChE↑, TAC↓, MDA↓, GSH↑	[129]
African sharpthooth catfish (*Clarias gariepinus*)	1.5 mg·L^−1^ CPF, *Spirulina platensis* and β-glucan (0.5%)	CPF: ALT↑, AST↑, LDL↑, Glucose↑, Triglycerides↑, MDA↑, antioxidants↓, P450↑ CPF + *Spirulina*: Phagocytic activity↑, P450↓, MDA↓	[130]
Nile tilapia (*Oreochromis niloticus*)	*CPF* (15 mg·L^−1^) *Chlorella vulgaris* (2–3%), 4/8 weeks	CPF: HSP70↑, GPx↑, GS↑, GR↓; IL-1β↑, TNF-α↑, TGFβ1↑, IL-8↑ CPF + *Chlorella*: HSP70↑↑, GPx↑↑, GS↑↑, GR↓↓; IL-1β↑↑, TNF-α↑↑, TGFβ1↑↑, IL-8↑↑	[131]
Mixed effects			
*Parachromis dovii*, *Poecilia gillii*	CPF (5 µg·L^−1^) + difenoconazole (325 µg·L^−1^)	CYP1A ↑, EROD↑, ChE ↓	[132]
Neotropical fish (*Rhamdia quelen*)	Imidacloprid (0.11 µg·L^−1^) + propoxur (0.039 µg·L^−1^), 96 h	AChE↓, GST~↓, CAT↓↑, SOD↑, ROS~↓, ASAP~, antagonistic effect	[133]
Pacu (*Piaractus mesopotamicus*)	endosulfan (1.1 μg·L^−1^) + lambda-cyhalothrin (0.7 μg·L^−1^), 96 h	white blood cells count↑, Lymphocytes↓, Eosinophils↑↑, AST↓, ALT↓. GST↑, GR~, GPx~, CAT↑, TBARS↑, antagonistic effect	[134]

**Abbreviations**: NPSH—non-protein thiols, TBARS—thiobarbituric acid-reactive products, ASA, ASAP—antioxidant ability, GST—glutathione transferase, CAT—catalase, GPx—glutathione peroxidase, GR—glutathione reductase, SOD—superoxide dismutase, MDA—malondialdehyde, LDH—lactate dehydrogenase, SDH—succinate dehydrogenase, ROS—reactive oxygen species, RNS—reactive nitrogen species, TAC—total antioxidant capacity, AChE—acetylcholine esterase, ACP—acid phosphatase, AST—aspartate transaminase, GS—glutathione synthase, ALT—alanine aminotransferase, ALP—alkaline phosphatase, GSHt—glutathione total, EROD—7-*ethoxy*-resorufin-O-deethylase, CbE—carboxyl esterase, CPF—chlorpyrifos, CYP—cypermetrin, GLY—glyphosate.

### 4.2. Pesticide-Induced Carbonyl Stress in Fish

Proteins are also a common target for pesticides’ adverse effects. When pesticides cause harm, protein carbonyls are said to rise in animal and human tissue. It is worth mentioning that current attention has shifted to the development of novel environmental and clinical biomarkers based on protein damage, such as protein adducts, protein carbonyls, and 8-hydroxy-2-deoxyguanosine (8-OHdG, a DNA oxidized nucleoside) [86,135]. Organophosphate, thiocarbamate, triazine, and tetrazine pesticides, as well as phenylurea and pyrethroid esters, have all been shown to cause protein oxidative damage in a variety of species, including water animals [136,137]. Exposure of *Channa punctata* to deltamethrin (0.75 μg·L^−1^), endosulfan (10 μg·L^−1^), and paraquat (5 mg·L^−1^) for 12 h–28 days, as well as chlorpyrifos (0.26 mg·L^−1^ and 0.52 mg·L^−1^) treatment of *Cyprinus carpio* for 96 h and 240 h, resulted in significant increases in protein carbonyls and 8-OHdG in the gills, kidney, brain, and liver [138,139]. Shorter exposure times resulted in the most pronounced induction, especially after 48 h [138]. After 14 days of exposure, chlorpyrifos and malathion were able to promote overproduction of methylglyoxal as a reactive carbonyl species in the zebrafish liver, even at low concentrations (0.1 and 5 μg·L^−1^, respectively) [115].

The mode of response observed in the laboratory was shown to be similar in the field. When comparing the gill and liver tissue of *Rita rita* and *Cyprinus carpio* fish from two natural pesticide pollution vicinities in India to the correspondent reference vicinities, a considerably higher amount of protein carbonyls was discovered [140]. Despite the promising results regarding the correlation of protein oxidative damage biomarkers with pesticide pollution risk to non-targets and/or outcome prognosis, a follow-up study focusing on organophosphates, particularly terbuthylazine, a widely used pesticide, is recommended to fill the gaps in the field of interest.

In most of the cases mentioned, increasing protein oxidative lesions interplayed with neurological and behavioral disorders, among them suppression of the acetylcholine esterase activity, muscarinic effect, swimming behavior, and locomotor performance not only in fish but also in humans [118,141]. It is highly likely that some of the neurobehavioral symptoms in animals exposed to organophosphate pesticides may be due to the formation of highly reactive protein carbonyls and the generation of oxidative stress.

### 4.3. Pesticide-Induced Nitrosative Stress in Fish

Reactive nitrogen species (RNS) are a type of nitrogen molecule that is closely linked to oxygen. RNS is primarily caused by the interaction of exogenously and endogenously produced nitric oxide (NO) with ROS (e.g., H_2_O_2_ and O_2_^−^^•^) and reductants like lithium aluminum hydride (LiAlH_4_) [142]. Nitroxyl anion (NO^-^) (produced from the reduction of NO), nitrogen oxides, nitrosonium cation (NO^+^), S-nitrosothiols (RSNO), and dinitrosyl iron complexes are all nitric oxide-derived molecules. NO is an intracellular messenger that controls a variety of physiological functions. However, because of its high reactivity with other free radicals, NO can be harmful under pathological conditions. When one electron is removed from NO, NO^+^ is formed. NO^+^ can form nitrosocompounds when it reacts with nucleophiles [143].

RNS are important in a variety of biological activities, including physiological control. NO has a role in fish immunological responses, much as it does in higher vertebrates [144]. The rainbow trout iNOS gene was sequenced in its entirety, and the gene organization was compared to that of human iNOS. While there are minor variations from the human gene, the exons demonstrate remarkable sequence and organization conservation [145]. In zebrafish injected with lipopolysaccharide, NO and ROS generation, as well as iNOS and COX2 protein levels and proinflammatory cytokines, increased, implying that these molecules play a role in antitoxin defense [146].

If RNS are created and present in excess amounts, they can be damaging to cells. RNS have pleiotropic effects on cellular targets, with effects persisting even after posttranslational modifications and interactions with ROS [143]. Through the development of nitrosative stress, RNS interact with many cellular components, resulting in cellular abnormalities, cell damage, and cell death [142]. Nitrosative stress is caused by the nitrosylation of key protein cysteine thiols (S-nitrosylation) and metallocofactors of proteins when NO or similar species are produced after exposure to certain xenobiotic agents, such as pesticides.

Almost every paper that has been written on alterations in oxidative/nitrosative stress parameters after the effects of commonly used pesticides includes a section relating to significant alteration of the iNOS pathway. However, there is no clear trend in the NO-related index variation in terms of adverse pesticide effects. Some findings emphasized the dose-dependent suppression of nitrite release, superoxide production by leucocytes, and various immunoresponses (e.g., phagocytic response, lymphocyte proliferation) in triazophos-exposed leukocytes of the freshwater teleost *Channa* punctatus at concentrations of 0.1, 0.5, and 1 µg·mL^−1^ for 48 h [147]. Malathion and chlorpyrifos, on the other hand, at concentrations of 50 µg·L^−1^ and 3 µg·L^−1^, respectively, induced NO in zebrafish liver after 14 days of exposure [115]. Similar results were revealed in atrazine- and chlorpyrifos-exposed carp as well as permethrin-exposed zebrafish when studying the increased mRNA expression of nitric oxide synthase (iNOS) and in parathion-treated rats (18 mg·kg^−1^), which were claimed to increase brain regional citrulline as an indicator of nitric oxide production (up to 40–47%) [148,149,150]. Furthermore, 7-nitroindazole (30 mg·kg^−1^) suppression of neuronal nitric oxide synthase resulted in a significant increase in cholinergic symptoms of parathion poisoning and death [150].

To put it concisely, there is currently insufficient knowledge on NO changes in lower-vertebrate tissue in response to pesticide impacts, the majority of which are immunotoxicants and endocrine disruptors and so could serve as a trigger of the NOS and NF-κB signaling pathway. We can infer that production of nitric oxide as a specific defense response when exposed to xenobiotic stimuli can help to support pesticide tolerance and withstand negative effects caused by pesticides based on existing results on NOS alterations in mammals following pesticide exposure, but further studies are needed [151,152].

### 4.4. Immunomodulatory and Inflammatory Effects of Organophosphate and Triazine Pesticides

Pesticides can cause immune-cell malfunction that can lead to immunosuppression or immunostimulation, which can contribute to inflammatory and allergic reactions, as well as autoimmune diseases. Almost all studies devoted to organophosphates immunomodulation have been conducted on rodents or human cell lines, with the majority of them focusing on lymphocyte subset proliferation and activity [153]. A few studies have looked at how environmental xenobiotics, particularly pesticides, affect the immune system and inflammation in fish. Organophosphates, such as chlorpyrifos, diazinon, and phosalone, among others, can affect molecules involved in innate and adaptive immunological responses in fish. Studies have revealed that depending on the pesticide, experimental design, and concentration, the immune system is activated and an inflammatory response triggered [154] or, conversely, the immune system’s activation and ability to make inflammatory mediators is inhibited [155].

In most fish, tetrameric immunoglobulin M (IgM) is broadly considered the most common serum immunoglobulin type [156]. IgM continues to make up the majority of all three immunoglobulins seen in fish and plays a role in both innate and adaptive immunity, as well as mediates interactions with self-antigens implicated in apoptotic cell clearance [157,158]. Although fish immunoresponses vary depending on the pesticide, exposure time, and concentration, the most consistent response of IgM levels to organophosphate pesticides appears to be suppression, particularly during acute exposure, as demonstrated in common carp (*Cyprinus carpio*), Nile tilapia (*Oreochromis niloticus*), and Chinese rare minnows [155,159]. This is mostly associated with alterations in macrophage inflammatory cytokines [160]. In this context, chlorpyrifos (0.051 mg·mL^−1^) has been found to reduce the concentration of IgM in the plasma of Nile tilapia (*Oreochromis niloticus*) [155]. Furthermore, chlorpyrifos lowered IgM levels in the blood and spleen of crucian carp, but raised them in the kidneys. Chlorpyrifos also enhanced the expression of IL-6, IL-8, and TNF-α in carp head, kidney, and spleen tissue, which was amplified when combined with the triazine herbicide atrazine [154]. Another study on the immunotoxicity of organophosphate pesticides using diazinon as the model showed a significant decrease in phagocytic index in juvenile Nile tilapia, although no changes in circulating IgM concentration were observed [161]. In addition, the exposure of *Clarias gariepinus* to ½ LC50 Roundup and ½ LC50 Stomp led to a prominent decrease in phagocytic activity in agreement with misbalance in antioxidants and significant histopathological alterations, namely, severe congestion in hepatoportal blood vessels, multifocal areas of coagulative necrosis invaded with numerous leukocytes and erythrocytes, and macrovesicular steatosis [162]. In our previous work, on the other hand, zebrafish retain their ability to activate immunological responses after long-term exposure to 15 or 500 μg·L^−1^ Roundup and 0.1 or 3 μg·L^−1^ chlorpyrifos, implying that immunosuppression is unlikely under these settings [19].

Regarding the effect of commonly used pesticides on the cytokines, it has been reported that 4 μg·L^−1^ chlorpyrifos contamination of largemouth bass (*Micropterus salmoides)* for 60 days and acute short-term exposure of zebrafish 96 hpf larvae to 100 or 300 μg·L^−1^ chlorpyrifos led to upregulated expression of proinflammatory genes (*TNF-α*, IL-1β, IL-6, *IL-8*, *IL-15*), downregulated expression of anti-inflammatory genes (*TGF-β1,* *IL-10)*, severe histopathological lesions, and downregulated expression of antioxidant-related genes. All these cellular events determined promotion of apoptosis through overexpression of *CASP3*, *CASP8*, *CASP9*, and *BAX* [117,118]. On the other hand, in vitro exposure of silver catfish monocytes to atrazine at 1 and 10 μg·mL^−1^ reduced mRNA levels of TNF-α and MPO (myeloperoxidase), but did not affect IL-1β [163]. Regarding the mechanism, exposure to organophosphates has been demonstrated to activate calcium-mediated p38-MAPK and ERK signaling, supporting an inflammatory stage through NF-κB activation and elevated levels of proinflammatory cytokines, such as TNF-α and IL-6 [164].

It is believed that the immunosuppressive effects of some pesticides may affect the ability of fish to tackle subsequent infections ands parasitic and viral diseases. However, not many publications have been devoted to this particular topic. Some of the findings elaborate on pyrethroids and dinitroanilines. In particular, rainbow trout exposed to pendimethalin and then to hematopoietic necrosis virus were shown to have increased expression levels of *TNF1*, *TNF2*, *TLR3*, *Il-1β*, and *IFN*, but the difference was more profound in fish pretreated with herbicide. Meanwhile, the only gene that was downregulated in the pesticide-infected group was *β-def*, and there was no difference after a single virus exposure [165]. In addition, significant impairment of the immune system was noted in Nile tilapia after chronic exposure to bifenthrin, which, in turn, significantly reduced the resistance of fish against *Aeoromans* infection [10]. Similar findings were obtained in subjects exposed to pyrethroid pesticides, which are known to reduce host defenses against infection and cancer [166].

These contentious and incomplete studies on fish immune and inflammatory responses to the deleterious effects of increasing pesticide pollution have the appeal of shedding light on these issues, raising awareness of the dependence between vulnerable immunity in exposed fish and the ability to withstand and survive additional stressors, such as infection.

### 4.5. Mitochondrial and Lysosome Toxicity

Mitochondria, which are important in cellular energy and ROS production, must be linked to pesticide-induced metabolic changes. Fish toxicity is strongly linked to cytotoxicity; however, total fish toxicity is stronger than cytotoxicity. Fish or cell toxicity, on the other hand, is unrelated to mitochondrial toxicity, implying that few chemicals have the same harmful mechanism and a huge portion of them have none [167]. In general, different classes of pesticides share relatively the same features of mitochondrial toxicity, among them significant swelling in mitochondria, low cytoplasm/mitochondria ratio, less dense matrix and vacuolated mitochondria with matrix disoriented cristae in histosections, a decrease in NADH cytochrome C reductase and succinate cytochrome C reductase in the mitochondrial respiratory chain, uncoupling of mitochondrial oxidative phosphorylation, and a decrease in mitochondrial transmembrane potential [168,169,170]. It is worth noting that there is a paucity of data on mitochondrial toxicity as a response to triazine pesticide exposure in fish, but studies on mammalian models suggest the same mechanism of mitochondrial toxicity observed for other pesticide classes. As an example, atrazine exposure resulted in downregulation of many OXPHOS subunits’ expression (e.g., mitochondrial transcription factor A and sirtuin 3) and affected biogenesis factor expression in HepG2 and L6 cells [168].

Mitochondria generate a lot of free radicals, which contribute to a lot of pathological and degenerative changes, particularly when an organism is being affected by chemical stressors. After being exposed to pyriproxyfen (0.001–10 μmol·L^−1^ for 16 h), male zebrafish showed a significant decrease in complex I/II respiratory control and Ca^2+^ uptake capability in brain tissue, as well as a significant increase in O_2_ radical dots revealed by the MitoSOX test [169]. A noticeable mitochondrial impairment in Ca^2+^ release could be linked to a decrease of mitochondrial membrane potential or pesticide-induced endoplasmic reticulum damage. This result is similar to what was shown in our previous experiments using chlorpyrifos- and malathion-treated zebrafish [115]. However, a prominent decrease in SDH was in good agreement with the accumulation of ROS and an increase in methylglyoxal (as a by-product of glycolysis) in zebrafish liver only after chlorpyrifos exposure [115]. SDH was lowered and LDH was raised after treatment with organophosphate insecticides, indicating that anaerobic metabolism was favored and aerobic pyruvate oxidation was restricted [115,171].

Malathion causes less noticeable alterations in fish mitochondrial respiration and OXPHOS than chlorpyrifos. Malathion at 7.30 mg·L^−1^ for 96 h, for example, had no effect on the mitochondrial function of tambaqui liver, with no alterations in the activity of any protein complexes [127]. In contrast, malathion compromises oxidative phosphorylation in *Oncorhynchus kisutch*, causing a 38% reduction in electron transport system respiration capacity and a 46% reduction in respiratory control rate, which may compromise mitochondrial ATP generation [172]. However, it did not affect the mitochondrial respiration of states 3 and 4 or the flow through complexes II and IV [172].

The main parts of reactive and specifically acting pesticides according to mode of action (MOA) classification can exert mitochondrial toxicity via electrophile–nucleophile interactions with biopolymers or specific interactions with certain receptor molecules [167]. It has recently been proved that mitochondrial translocator protein binding affinity, mitochondrial membrane potential reduction in HepG2 cells, and developmental toxicity in zebrafish are all substantially linked with organophosphate pesticide (e.g., chlorpyrifos, azinphos-methyl phosalone, malathion, mevinphos) hydrophobicity [17]. Moreover, Leung and Meyer emphasized that energy metabolism could be a site of harmful action for some organophosphate pesticides, especially for those compounds that have similarities to Krebs cycle intermediates (e.g., malathion and mevinphos) [17]. The findings also support the theory that some organophosphate pesticides, particularly those that need enzymatic activation to the oxon form, may have mitochondrial effects in addition to the known consequences of cholinergic transmission disruption [17].

The functionalities of mitochondria and lysosomes are proven to be coordinated with cellular metabolism and signaling [173]. In the breakdown and degradation of old organelles and damaged proteins, the lysosomal system is crucial. Chaperone-mediated autophagy, macroautophagy, and endocytosis convey misfolded and damaged proteins to lysosomes and then pursue degradation by the ubiquitin–proteasome pathway [174,175]. Meanwhile, proteostasis is disrupted, endoplasmic reticulum stress is increased, and autophagy is inhibited when the lysosomal system is damaged or dysfunctional [176]. In vertebrates, such as fish and mammals, lysosomal dysfunction is frequently linked to pesticide-induced cytotoxicity [115,177,178]. In zebrafish, both in vitro and in vivo, novel metal insecticide [Mg(hesp)_2_(phen)] (10 and 1000 ng·mL^−1^), chlorpyrifos (0.1 and 3 μg·L^−1^), carbofuran (0.1, 0.05 and 0.02 μg·L^−1^ respectively), and Roundup (67.7–270.8 and 500 μg·L^−1^ respectively) can cause lysosomal membrane instability, as evaluated by neutral red retention and activity of a critical acidic lysosomal protease, cathepsin D, which increased by ~27% when compared with the correspondent control [19,178,179].

Considering all of this evidence, it allows us to suggest a pivotal role of mitochondria and highly likely a pertinent role of lysosomes for the adaptation of organisms to pesticide pollution. The preserving of mitochondrial function and normal metabolic activities is an attempt to compensate for the contaminants’ harmful consequences.

### 4.6. Pesticide-Induced Endocrine Disorders

Pesticides that might interfere with hormone-related pathways have been linked to negative impacts on both the human and animal reproductive systems [180]. About 146 substances, among them pesticides, are included in the PAN-EU list of chemicals that provoke endocrine disruption [181], but in fact, a lot more pesticides might affect hormone-dependent pathways as they are able to perform as endocrine disruptors. They can alter sex-steroid levels, induce reproductive behavioral changes, perturb steroid synthesis, affect gonadal morphology, and provoke developmental toxicity in several fish species [182].

The induction of vitellogenin (Vtg) protein in male fish has been extensively employed as a biomarker for exposure to xenoestrogens. In fish, Vtg can be defined as a lipoglycophosphoprotein that is synthesized in the liver, released into the bloodstream, and acts as a key precursor of the proteins found in egg yolks. In general, despite having VTG genes, male specimens have very low or no Vtg levels [183]. When exposed to specific synthetic estrogens, alkyl phenols, phytoestrogens, and pesticides, Vtg synthesis in male specimens increases significantly, and Vtg can be detected in blood or hepatocytes [184]. Vitellogenin levels were claimed to be higher in carp blood serum after exposure to Apollo (Clofentezine, 1 and 2 μg·L^−1^) and Tattu (mancozeb, 9.1 and 91 μg·L^−1^) for 14 days [185]. Although it has recently been reported that chlorpyrifos at a concentration range of 0.25–3.02 μM was not endocrine-active and had no potential to interact with the estrogen, androgen, or thyroid pathways based on weight-of-evidence evaluation in fathead minnow [186], several studies proposed the opposite results. It is worth noting that chlorpyrifos at concentrations of 0.174 μmol·L^−1^ (for 96 h), 3 μg·L^−1^ (for 14 days), and 20 μg·L^−1^ in catfish, zebrafish, and rainbow trout altered progesterone receptors, affected vitellogenin levels in liver, ovary, and serum, and induced steroid hormones, namely, estradiol, progesterone, and maturation-inducing hormone [19,187,188]. Similar observations were found for other organophosphate pesticides, including glyphosate, diazinon, and monocrotophos [19,189,190,191,192]. In particular, exposure of carp males to 0.488 and 0.976 mg·L^−1^ diazinon for 15 days reduced E2 and plasma Vtg levels alongside with degeneration, congestion, and fibrosis in testis [191]. Long-lasting exposure (30 days) caused total disruption in Vtg synthesis in close linkage with profound histopathological changes in hepatocytes and gonads [191].

Tetrazine pesticides have a quite similar effect on vitellogenesis and reproductive function in fish to organophosphates. Atrazine and simazine are noted to be able to increase estrogen production, aromatase activity, and zcyp19a1 expression (target gene of the nuclear receptor SF-1), alter steroid hormone metabolism and vitellogenesis, disrupt the hypothalamic control of luteinizing hormone and prolactin levels, and promote morphological changes in specimens who are being exposed [193,194,195]. Atrazine exposure at a concentration of 1000 µg·L^−1^ for 7–21 days led to a decrease in plasma testosterone and estradiol, but did not affect vitellogenin in *Carassius auratus* males [193]. It is highly likely that vitellogenesis is not a major priority target for organophosphate pesticides, but it can affect it, particularly in the case of coexposure [19,187].

The CYP19 gene encodes aromatase, an enzyme that changes testosterone into estradiol. Using knockout zebrafish lines *cyp19a1a^−/−^* (only male specimen), it was proved that aromatase plays a critical role in directing ovarian differentiation and development and controls physiological functions of estrogens in fish [196]. Aromatase is also involved in management of numerous physiological processes related to reproduction and maintaining the masculinization of male sexual behavior. Zebrafish exposed to 0.001, 0.010, and 0.100 mg·L^−1^ 40% monocrotophos for 40 days showed significant increases in the expression of both gonadal and brain aromatase (CYP19a1a, CYP19a1b), forkhead transcription factor (FOXL2), and proportion of females (71%), as well as suppression of doublesex/mab-3 related transcription factor 1 (DMRT1) [197]. Organophosphate and triazine pesticides are considered to affect aromatase activity, playing the role of allosteric activators or triggers of cytochrome P450 reductase, an effector on aromatase conformation [198]. Partial and mild inhibition of aromatase in MCF-7 cells by glyphosate, which is highly dependent on substrate concentration, has been demonstrated. Additionally, the inhibition was discovered to be noncompetitive at the lower glyphosate concentration (1 μM) examined; however, at the higher concentration (5 μM), the inhibition changed into a mixed inhibition mode [198]. Meanwhile, chlorfenvinphos, chlorpyrifos, and tetrachlorvinphos belong to the pesticides that were out of suppressing aromatase in JEG-3 cells [199]. Although being superficially observed in fish, aromatase and CYP19 genes need to be further studied regarding possible affection by organophosphate and triazine pesticides. Furthermore, using teleosts to delve deeper into the mechanisms and outcomes of endocrine disruptor pesticides may be more meaningful and efficient than in vitro studies using human cell lines.

A wide spectrum of pesticides, including organophosphates and triazines, may influence thyroid homeostasis. Antrazine, malathion, and chlorpyrifos may have an impact on thyrotrope synthesis, resulting in thyreotropine-lowering effects [180]. Through a thyroid hormone-dependent mechanism, chlorpyrifos prevents surgeonfish from reaching sexual maturity [200]. The treatment of goldfish with monocrotophos in the concentration range of 0.01–1.00 mg·L^−1^ for 21 days led to a significant decrease in plasma T3 and alteration in expression patterns of the hypothalamic–pituitary–thyroid (HPT) axis-responsive genes [201]. On the contrary, environmental realistic and occupational concentrations of organophosphate and triazine pesticides can simulate triiodothyronine synthesis, as demonstrated in yellow-tailed tetra fish (*Astyanax altiparanae*) after 10 μg·L^−1^ atrazine for 30 days, zebrafish after 0.1 and 3 μg·L^−1^ chlorpyrifos for 14 days, and a murine model after short-term low-dose chlorpyrifos exposure alongside significant thyroid tissue damage appearing as irregular contours of thyroid follicles and few or no colloids [19,99,202]. It is interesting that an increase in total testosterone and T3 was observed to interact with diethylthiophosphate, a nonspecific metabolite of organophosphate pesticides [203]. The modulation of T4 production in the thyroid gland, elevation of UDPGT and SULT, enzymes involved in the hepatic metabolism of thyroid hormones, and promotion of extrathyroidal conversion of T4 to T3 catalyzed by deiodinase are all potential mechanisms underpinning the hypothyroid-like impact of organophosphates [203]. In addition, organophosphates are able to dock into membrane thyroid hormone receptor by molecular docking, thereby supporting the competitive binding experiment. The quantitative proteomic study found that tris(1,3-dichloro-2-propyl) phosphate caused improper protein expression in zebrafish embryonic development. These protein interactions may be connected to membrane thyroid hormone receptor, which, in turn, might be an important mediator of the thyroid hormone-disrupting effects of organophosphates [204].

The latest findings highlight that in zebrafish, atrazine affects gene expression relating to endocrine disruption much more significantly than chlorpyrifos. Meantime, glucokinase, which acts as a glucose sensor and is linked to diabetes and hypoglycemia, was reported to be among the genes dysregulated by both pesticides [205]. It is worth mentioning that pesticide-related stimulation of the cholinergic receptors and nicotinic acetylcholine receptors may lead to disturbances in insulin and glucagon secretions in animals and humans [206]. Although fish insulin was one of the first vertebrate insulins isolated and piscine models have become the most widely used for studying pancreatic hormone generation and processing [207], mechanistic studies of pancreatic hormone responses to organophosphate and triazine pesticides have been beyond the scope of research. Few reports have disclosed the negative impact of organophosphate pesticides on pancreatic zymogen granule stores in fish or variation in the expression of genes involved in glucose metabolism in the liver and depletion of glycogen stores [205,208]. On the other hand, information available regarding people who are permanently exposed to organophosphate pesticides via different routes allows us to conclude a potential link between organophosphate-mediated injury to pancreatic beta cells, increased hepatic gluconeogenesis, and systemic inflammation on the one hand and diabetes mellitus on the other [209]. However, delving deeply into the pancreatic toxicity of pesticides is urgently needed, particularly using piscine as the model for mechanistic studies because of the similarity of the responses to higher vertebrates [207].

To conclude, the published research provides some evidence that the environmentally relevant concentrations of organophosphates and triazine pesticides may well act as endocrine disruptors and affect the corticosteroid status, pancreatic hormones, and thyroid equilibrium in fish. However, the mode of action remains to be clarified. In addition, a broader spectrum of pesticides has to be studied because of insufficient data regarding widely used pesticides that might affect non-targets.

## 5. Conclusions

**Figure 2:**
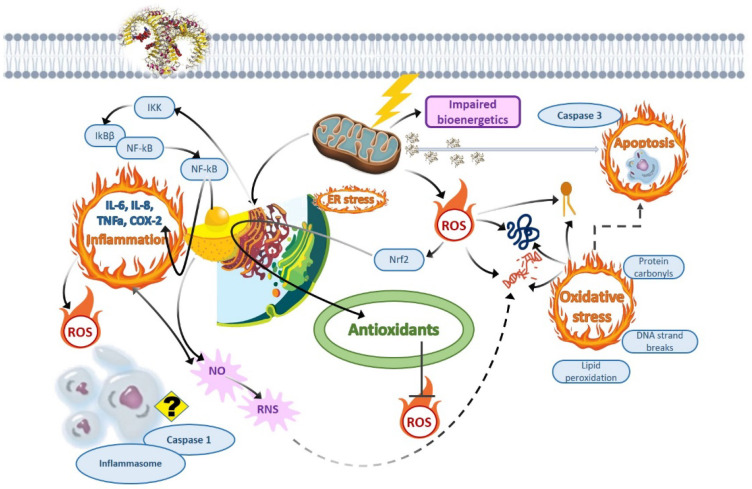
Schematic representation of the potential cellular and molecular events in fish hepatocytes under the action of organophosphate and triazine pesticides.

Taken together, organophosphates (e.g., chlorpyrifos, glyphosate), phenylureas (e.g., chlorotoluron), triazines (e.g., terbuthylazine), and neonicotinoids belong to the most commonly used and detected pesticides in water bodies all over the world, including Central and Eastern Europe. Fish can accumulate organophosphate and triazine pesticides and their residues more efficiently than mollusks. Once accumulated, organophosphate and triazine pesticides may affect cell redox homeostasis and induce immunotoxicity, neuroendocrine disorders, and cytotoxicity, which are manifested in oxidative stress, lysosomal and mitochondrial damage, inflammation, and apoptosis/autophagy (Figure 2).

Based on our rigorous analysis, we identified knowledge gaps in the literature for figuring out potential negative effects of pesticides on fish and their processes. Freshwater fish have been the focus of the majority of fish research. An effort should be made to examine responses in marine fish, especially in the case of a mechanistic approach, keeping in mind species specificity and salinity regime, which may affect bioaccumulation. Not much is known about the effects of triazine herbicides, particularly terbuthylazine and simazine, on biochemical traits of non-targets, despite the fact that these substances are widely used and capable of causing profound damaging effects. All reported studies in humans or animals support the idea that pesticides promote ROS and RNS overproduction, which in turn reveal oxidative damage of lipids, proteins, and DNA. Despite the fact that NO (iNOS) pretends to play a dual role in protecting rodents from pesticide exposure, putative NO roles in fish have received little attention. Inflammasome activation and pyroptosis via mitochondrial oxidative stress may be alternative mechanisms of harmful effects of organophosphate pesticides in fish. At least 50% of the research has been done to evaluate the acute short-term adverse effects of pesticides on non-targets. More emphasis should be given to this field of research, since fish are exposed to pollutants in environmentally or occupationally relevant concentrations, but not acute ones. Long-term impacts should also be taken into consideration, because pesticides may increase the sensitivity of the organism to a later occurrence, incurring a tolerance and fitness cost, which might only be seen when an additional stressor appears or later in life. Few studies address mixture effects, despite the fact that they accurately reflect natural environment scenarios. A global strategy might be developed to address the issue of highly hazardous pesticides in the context of developing low-cost and highly effective risk-assessment protocols for identifying pesticides that pose the greatest risk to human health. As feasible mitigation measures, the synthesis of biomarker-based risk assessment of novel, alternative, less hazardous pesticide formulations must be made a reality.

## Data Availability

Not applicable.

## Abbreviations

AChE	acetylcholinesterase
ALP	alkaline phosphatase
ALT	alanine transaminase
AMPA	aminomethylphosphonic acid
AST	aspartate transaminase
BAF	bioaccumulation factor
BCF	bioconcentration factor
CAT	catalase
GOT	glutamic oxaloacetic transaminase
GPT	glutamic pyruvate transaminase
GPx	glutathione peroxidase
GST	glutathione s-transferase
HQ	hazard quotient
*K*_ow_	n-octanol–water partition coefficient
LDH	lactate dehydrogenase
LOQ	limit of quantification
LPO	lipid peroxidation
PCBs	polychlorinated biphenyls
MDA	malondialdehyde
RNS	reactive nitrogen species
ROS	reactive oxygen species
SOD	superoxide dismutase
TBARS	thiobarbituric acid-reactive substance
